# Racial and ethnic disparities in obesity and contributions of social determinants of health among boys with autism spectrum disorder

**DOI:** 10.3389/fped.2023.1198073

**Published:** 2023-07-11

**Authors:** Sandy Magaña, Misha Eliasziw, April Bowling, Aviva Must

**Affiliations:** ^1^ Steve Hicks School of Social Work, University of Texas at Austin, Austin, TX, United States; ^2^ Department of Public Health & Community Medicine, Tufts University School of Medicine, Boston, MA, United States; ^3^ Department of Nursing and Health Sciences, Merrimack College, Andover, MA, United States; ^4^ E.K. Shriver Center, UMASS Chan Medical School, Worcester, MA, United States

**Keywords:** obesity, weight status, race, ethnicity, health disparities, autism spectrum disorder, social determinants of health

## Abstract

Children with autism spectrum disorders (ASD) are at greater obesity risk compared to typically developing peers. Although many potential risk factors for this relationship have been identified, the causal chain must be better understood, particularly modifiable social determinants of obesity risk in ASD, and especially for children with ASD from minoritized racial/ethnic groups. We aimed to: (1) examine racial/ethnic disparities in obesity status in boys with ASD; (2) assess associations between social determinants of health and obesity status; and (3) understand if social determinants of health factors mediate the relationship between race/ethnicity and obesity status for these youth. We used data for 124 boys, aged 9–10 with ASD enrolled in an ongoing longitudinal study. Social determinants of health explored included socioeconomic position, Area Deprivation Index, neighborhood safety, food and housing insecurity, and racial/ethnic discrimination. The racial/ethnic distribution was: 17.1% Black, 14.6% Latino, and 68.3% White; average age was 10 years. Both Black (PR 2.57, 95% CI: 1.26–5.26) and Latino boys (PR 2.08, 95% CI: 1.08–4.03) with ASD were more likely to be obese than their White peers. While there were significant differences in some social determinants of health by race/ethnicity, only food insecurity mediated associations between race/ethnicity (Black vs. White) and obesity. The striking disparities in obesity and differences in social determinants of health between Black and Latino children with ASD compared to White children emphasize the need to identify factors that contribute to healthy weight among these children and to address these factors in practice.

## 1. Introduction

Obesity prevalence is high among children, and puts them at risk for myriad poor physical health outcomes including Type 2 diabetes, hypertension, and orthopedic conditions (1). Childhood obesity has also been associated with mental health risks, such as decreased social support and depression (2, 3). These health outcomes are linked to the development of chronic disease in adulthood (1, 4). At the same time, an emerging body of research suggests that children with autism spectrum disorders (ASD) have higher prevalence of obesity compared to typically developing (TD) children; for example, a recent meta-analysis found an increased risk of obesity among children with ASD compared to their TD peers (5). Differing associations with obesity by sex or gender among youth with ASD are likely, as are observed among TD youth (6). However, because approximately four males are diagnosed with ASD for every female diagnosed (7), most studies to date have been underpowered to examine moderation by sex and have relied on statistical adjustment to control for sex differences. This is problematic, as such adjustment provides a summary estimate and may mask important differences in the magnitude or direction of associations.

There are a multitude of factors that have been shown to contribute to higher rates of obesity among children with ASD. For example, screen time is higher among youth with ASD (8), and physical activity lower compared to TD youth (9) due to a variety of barriers (10) including a lack of inclusive physical education, sports, and community recreation programs (11). Children with ASD are more likely to display selective eating patterns, which can result in low fruit and vegetable consumption, eat more foods that are high calorie and have low nutrient density, and have higher intake of sugar sweetened beverages (12–14). Importantly, psychotropic medications used to treat many children with ASD, such as second-generation antipsychotics, are associated with significant, and often rapid weight gain (15).

A variety of social determinants of health may affect obesity risk among children with ASD. For example, perceived discrimination and decreased social support have been shown to be associated with increased adiposity in TD youth (16) and both of these domains are elevated in populations with ASD (17). Also, well established social determinants of health, such as socioeconomic status, food insecurity, and housing insecurity maybe more prevalent among families of autistic children, given the increased resource demands they face, such as caregiving and medical costs. For example, Karpur et al. found that households that included a child with ASD had twice the risk of food insecurity of households with TD children only (18). However, to our knowledge, no study to date has investigated whether these social determinants are associated with obesity in children with ASD.

Importantly, there are established disparities in obesity for Black and Latino TD children compared to non-Latino White TD children which are related to social determinants of health. By adolescence, prevalence of overweight and obesity in the United States is approximately 9% higher among African American boys and 14% higher among Latino boys relative to their white counterparts (19). These disparities emerge in early childhood, increase over time (19), and are particularly pronounced in the southeastern United States (20). Important contributors to these disparities include gaps in socioeconomic status, accessibility and affordability of healthy foods, and access to green, safe, and walkable neighborhood environments (21). However, there is also emerging evidence that prenatal adversity (22), high allostatic load, and perceived discrimination in childhood may contribute to obesity development in childhood, particularly among African American and Latino youth whose parents were not US-born (23).

The research on obesity among racial and ethnic minoritized children with ASD is sparse but suggestive of poor outcomes compared to their counterparts with ASD and non-Latino white children. Data from the National Health Interview Survey found that Black adolescents with ASD had a higher risk than their same-race counterparts without ASD (24). Similarly, A meta-analysis found that non-White children with ASD had higher obesity rates than their typically developing counterparts, while this was not true for non-Latino White children (5). A study using data from the Autism Treatment Network found that Latino children had a higher risk of obesity compared to non-Latino White children with ASD (25). The intersection between ASD and minoritized status when studying childhood obesity is important. In addition to the factors that contribute to obesity among children with ASD, Black and Latino children with ASD are likely to face additional factors associated with social determinants of health, as they are more likely to live in poverty, have higher barriers to physical activity and health eating, and have increased risk of social isolation and perceived discrimination.

To address this gap in the literature, we analyzed a national sample of boys who were of the same ethnicity and race as their parent to address the following research questions: (1) Are there racial/ethnic disparities related to weight status for Black and Latino boys with ASD compared to non-Latino White boys?, (2) What social determinants of health are associated with weight status among boys with ASD?, and (3) Do social determinants of health mediate the relationship between race/ethnicity and weight status?

## 2. Methods

### 2.1. Study population

We used data from the Adolescent Brain Cognition Development (ABCD) Study, an ongoing longitudinal study of 9 and 10 year-old children. The baseline sample was recruited from 21 study sites from September 2016 to October 2018. At each study site, consenting parents and their assenting children were recruited largely from public and private schools. Eligibility criteria required that children be attending regular (mainstream) classes in school. The geographic locations comprising the ABCD research sites are nationally distributed and generally represent the range of demographic and socio-economic diversities in the United States. Information about the sample design, recruitment, measures, and compensation is detailed elsewhere (26, 27).

### 2.2. Measures

#### 2.2.1. Autism status

We included only participants with ASD, defined based on a single question on the screening questionnaire, by parents answering yes to the following question: “Has your child been diagnosed with autism spectrum disorder?”

#### 2.2.2. Weight status

Body mass index (BMI) was based on measures of height and weight, which were taken as the average of up to 3 separate measurements. BMI was calculated as weight in kg divided by height in meters squared. Sex and age-specific BMI z-scores (BMIz) were referenced against the Center of Disease Control 2001 (28). BMIz scores were used to define obesity (≥95th percentile) or not (<95th percentile).

#### 2.2.3. Demographic characteristics

Parents answered questions about their age, gender, birth country (outside US versus US born), marital status, number of individuals living in the household, as well as their child's age and gender. Parent-reported ethnicity and races were categorized as Latino, non-Latino White, non-Latino Black, non-Latino Asian, and non-Latino other/multi-racial.

#### 2.2.4. Social determinants of health

Individual socioeconomic position (SEP) scores were calculated from a weighted combination of four highest household education levels and four household income levels, combining education and income into a socioeconomic position score for use in studies of health inequalities (29, 30). The SEP scores range from 1 to 10, with higher scores corresponding to higher socioeconomic positions. An area deprivation index (ADI) was calculated for each household that measures socioeconomic disadvantage from a composite of 17 census variables that include education, income, employment, housing, and household characteristics (30). The ADI scores are percentiles that range from 0 to 100, with higher scores corresponding to higher levels of area deprivation. Neighborhood Safety was measured by 3 yes/no questions to parents: I feel safe walking in my neighborhood, day or night; violence is not a problem in my neighborhood; and my neighborhood is safe from crime. Food and housing insecurity was measured by two yes/no questions to parents asking if there was time in the past 2 months when the family couldn't afford food, or to pay rent or mortgage. Racial/ethnic discrimination was assessed by a single yes/no question.

#### 2.2.5. Statistical analyses

Bivariable analyses consisted of comparing continuous variables among the three race and ethnic groups using analyses of variance and comparing categorical variables using chi-square tests. Generalized linear models using generalized estimation equations were used to derive the estimates and inferences for the BMIz scores and obesity analyses. An autoregressive (1) correlation structure was used to account for the repeated BMIz and weight status measures over time, as well as including the primary parent's and son's baseline ages as covariates in the models. For analyzing BMIz, a normal distribution with an identity link was specified in the models. Adjusted mean differences with corresponding 95% confidence intervals were used to compare the three groups. For analyzing obesity, a binomial distribution with a log link was specified in the models. Adjusted prevalence ratios with corresponding 95% confidence intervals were used to summarize the results. Mediation analyses were conducted to determine the extent that social determinants of health (ADI, neighborhood safety, food and housing insecurity, and racial/ethnic discrimination) explains (i.e., mediates) the relationship between measures of weight status and the three categories of race and ethnicity. For BMIz, the percentage mediated was calculated as the relative difference between the unadjusted and adjusted mean differences. For obesity, the percentage mediated was calculated as the relative difference between the unadjusted and adjusted prevalence ratios. All statistical analyses were carried out using SAS 9.4 (SAS Institute Inc., Cary, NC), and results with *p*-values < 0.05 deemed statistically significant.

## 3. Results

The ABCD dataset consisted of 11,876 individuals, of which 201 (1.7%) reported their “child been diagnosed with autism spectrum disorder”. Four individuals were excluded because of missing age, gender, ethnicity, or race data, and 13 were excluded because the area deprivation index percentile was missing. Among the 184 children with ASD, 158 (85.9%) were males, of which 123 (77.8%) had the same racial and ethnic identity as their primary parent. The racial and ethnic distribution of the parent-son dyads was: 21 (17.1%) Black, 18 (14.6%) Latino, and 84 (68.3%) White. Given that families were followed longitudinally, annual measurements of weight and height were available from a majority of children. Black children contributed 56 measurements, Latino children contributed 45 measurements, and White children contributed 217 measurements to the BMIz and obesity analyses.

Demographic characteristics of the dyads are reported in Table 1. The mean (standard deviation) age of the children at baseline was 10.0 (0.6) years. Black and Latino parents were significantly less educated and financially poorer than White families. These observations are supported by significantly lower mean socioeconomic position scores and higher ADI percentiles among Black and Latino families (*p*-value < 0.001). No child reported experiencing discrimination based on their race or ethnicity.

**Table 1:** Sociodemographic characteristics and social determinants of health by race and ethnicity.

	Black (*n* = 21)	Latino (*n* = 18)	White (*n* = 84)	*P*-value
Sociodemographic variables				
Age of child (years), mean (standard deviation)	10.2 (0.5)	9.9 (0.5)	9.9 (0.6)	0.12
Age of primary parent (years), mean, (standard deviation)	38.7 (8.2)	39.3 (7.9)	41.8 (6.3)	0.11
Gender of primary parent, *n* (%)				
Female	18 (85.7)	16 (88.9)	79 (94.0)	0.40
Male	3 (14.3)	2 (11.1)	5 (6.0)	
Birth country of primary parent, *n* (%)				
United States	19 (90.5)	9 (50.0)	83 (98.8)	<0.001
Outside United States	2 (9.5)	9 (50.0)	1 (1.2)	
Marital status, *n* (%)				
Not married nor partnered	11 (52.4)	7 (38.9)	19 (22.6)	0.02
Married or partnered	10 (47.6)	11 (61.1)	65 (77.4)	
Number living in household, median, (interquartile range)	5 (4–6)	4 (3–5)	4 (4–5)	0.02
Highest household education, *n* (%)				
Grade school	1 (4.7)	0 (0.0)	0 (0.0)	< 0.001
High school	6 (28.6)	7 (38.9)	5 (5.9)	
College or vocational	6 (38.6)	5 (27.8)	24 (28.6)	
University or postgraduate	8 (38.1)	6 (33.3)	55 (65.5)	
Highest household employment status				
Not working	9 (42.9)	4 (22.2)	11 (13.2)	0.03
Part-time working	1 (4.8)	0 (0.0)	5 (5.9)	
Full-time working	11 (52.4)	14 (77.8)	68 (80.9)	
Household income, *n* (%)				
<$50,000	15 (71.4)	12 (66.7)	24 (28.6)	0.004
$50,000–$74,999	1 (4.8)	1 (5.5)	4 (4.8)	
$75,000–$99,999	0 (0.0)	0 (0.0)	15 (17.9)	
$100,000 or greater	4 (19.0)	3 (16.7)	37 (44.0)	
Refused to answer	1 (4.8)	2 (11.1)	4 (4.7)	
Socioeconomic position score, mean (standard deviation)	5.0 (3.1)	4.8 (3.0)	7.5 (2.7)	<0.001
Socioeconomic position score 4 or lower, *n* (%)	13 (61.9)	11 (61.1)	19 (22.6)	<0.001
Area Deprivation Index percentile, mean (standard deviation)	69.3 (32.9)	39.9 (24.2)	31.4 (22.1)	<0.001
Area Deprivation Index at 85th percentile or higher, *n* (%)	11 (52.4)	1 (5.6)	3 (3.6)	<0.001
National Walkability Index, mean (standard deviation)	12.3 (4.4)	12.1 (3.9)	10.1 (3.8)	0.02
National Walkability Index 10 or lower, *n* (%)	6 (28.6)	5 (27.8)	49 (58.3)	0.008
Neighborhood safety				
I feel safe walking in my neighborhood, day or night, *n* (%)	14 (66.7)	12 (66.7)	66 (78.6)	0.37
Violence is not a problem in my neighborhood, *n* (%)	10 (47.6)	12 (66.7)	61 (72.6)	0.09
My neighborhood is safe from crime, *n* (%)	8 (38.1)	10 (55.6)	49 (58.3)	0.25
Food and housing security				
In the past 12 months, there has been a time when the immediate family needed food but couldn’t afford to buy it or couldn't afford to go out to get it, *n* (%)	4 (19.1)	3 (16.7)	8 (9.5)	0.40
In the past 12 months, there has been a time when the immediate family didn’t pay the full amount of the rent or mortgage because they could not afford it, *n* (%)	5 (23.8)	3 (16.7)	11 (13.1)	0.47
Discrimination				
In the past 12 months, I have felt discriminated against because of race, ethnicity, or color, *n* (%)	0 (0.0)	0 (0.0)	0 (0.0)	1.00
Total visits contributed to weight analyses, *n*	56	45	217	
Baseline, *n* (%)	21 (37.5)	18 (40.0)	84 (38.7)	0.98
1-year follow-up, *n* (%)	21 (37.5)	17 (37.8)	76 (35.0)	
2-year follow-up, *n* (%)	14 (25.0)	10 (22.2)	57 (26.3)	

To answer research question 1, we examined BMIz scores and obesity by race and ethnicity. Although mean BMIz scores were significantly different among the three racial/ethnic groups (*p*-value = 0.02), the observed differences were primarily in comparison to White children (Table 2). Mean differences between Black and Latino children were non-significant. A similar pattern was observed for the obesity results (Table 2). Both Black and Latino children were significantly more than twice as likely to be obese compared to White children.

**Table 2:** Body mass index and obesity status by race and ethnicity.

	Black (*n* = 56)	Latino (*n* = 45)	White (*n* = 217)	*P*-value
BMIz, mean (se)	1.17 (0.31)	1.04 (0.25)	0.38 (0.14)	0.02
Mean difference	0.79	0.66	Reference	
95% confidence interval	(0.08–1.49)	(0.09–1.24)		
*P*-value	0.03	0.02		
Obese, *n* (%)	21 (40.3)	15 (32.6)	36 (15.7)	0.02
Prevalence ratio	2.57	2.08	Reference	
95% confidence interval	(1.26–5.26)	(1.08–4.03)		
*P*-value	0.01	0.03		

Estimates and *p*-values were derived from generalized linear models using generalized estimating equations and adjusted for primary parent's and son's baseline ages.

To answer research question 2, we examined whether social determinants of health were associated with BMIz and with obesity (Table 3). Socioeconomic position and the ADI were not significantly associated with BMIz despite the mean BMIz scores being almost twice as high among disadvantaged families. Consistent with the BMIz findings, prevalence of obesity almost twice as likely among disadvantaged families (SEP *p* = 0.05 and ADI *p* = 0.04). None of the neighborhood safety measures were associated with either BMIz or obesity. Food insecurity and housing insecurity were significantly associated with both BMIz and with obesity; food insecurity was associated with a more than 3-fold increase in obesity prevalence and housing insecurity with a nearly 2-fold increase.

**Table 3:** Association of social determinants of health with BMIz and with obesity.

	BMIz mean and mean difference (*n* = 318)	*P*-value	Obesity prevalence and prevalence ratio (*n* = 318)	*P*-value
Socioeconomic position score 4 or lower (vs 5 or higher)	0.91 vs 0.45 0.46	0.08	0.32 vs 0.18 1.78	0.05
Area Deprivation Index at 85^th^ percentile or higher (vs lower than then 85^th^)	0.98 vs 0.56 0.42	0.24	0.39 vs 0.20 1.94	0.04
National Walkability Index 10 or lower (vs 11 or higher)	0.52 vs 0.70–0.18	0.46	0.25 vs 0.20 1.27	0.43
I feel safe walking in my neighborhood, day or night (vs not)	0.59 vs 0.68–0.09	0.77	0.22 vs 0.22 1.00	0.98
Violence is not a problem in my neighborhood (vs not)	0.60 vs 0.64–0.04	0.87	0.19 vs 0.28 0.69	0.22
My neighborhood is safe from crime (vs not)	0.61 vs 0.62–0.01	0.97s	0.22 vs 0.23 0.96	0.89
In the past 12 months, there has been a time when the immediate family needed food but couldn't afford to buy it or couldn't afford to go out to get it (vs not)	1.52 vs 0.48 1.04	0.006	0.58 vs 0.18 3.26	< 0.001
In the past 12 months, there has been a time when the immediate family didn't pay the full amount of the rent or mortgage because they could not afford it (vs not)	1.15 vs 0.51 0.64	0.03	0.38 vs 0.19 1.96	0.03

Estimates and *p*-values were derived from generalized linear models using generalized estimating equations and adjusted for primary parent's and son's baseline ages.

To address research question 3, Table 4 presents the results of the mediation analyses, which quantify the extent that each social determinant of health explains the association between race/ethnicity and BMIz and obesity for those determinants that were significantly associated with weight outcomes. In general, the social determinants of health were found to be weak mediators. Only food insecurity reached the level of being a partial mediator, explaining approximately one-quarter of the association between race/ethnicity and obesity.

**Table 4:** Percentage of BMIz and obese relationships with race and ethnicity mediated by selected social determinants of health.

	BMIz Black vs. White	BMIz Latino vs. White	Obese Black vs. White	Obese Latino vs. White
Socioeconomic position score 4 or lower (vs. 5 or higher)				
Percentage mediated	11.3%	12.2%	18.7%	13.3%
*P*-value	0.14	0.24	0.20	0.10
Area Deprivation Index at 85th percentile or higher (vs. lower than then 85th)				
Percentage mediated	0.0%	0.0%	0.0%	0.0%
*P*-value	1.00	1.00	1.00	1.00
In the past 12 months, there has been a time when the immediate family needed food but couldn’t afford to buy it or couldn’t afford to go out to get it (vs. not)				
Percentage mediated	5.9%	6.5%	29.1%	22.7%
*P*-value	0.18	0.25	0.02	0.18
In the past 12 months, there has been a time when the immediate family didn’t pay the full amount of the rent or mortgage because they could not afford it (vs. not)				
Percentage mediated	6.7%	0.4%	6.2%	15.1%
*P*-value	0.19	0.42	0.13	0.42

Estimates and *p*-values were derived from generalized linear models using generalized estimating equations and adjusted for primary parent's and son's baseline ages.

## 4. Discussion

In this paper, we examined racial and ethnic health disparities among boys with ASD in relation to an important health outcome for children: obesity. Health disparities are differences in health-related outcomes and services that are associated with social and economic disadvantage (31). It is important to identify such disparities in order to take actions to increase health equity for all. Using a US-based national sample, we found striking disparities in obesity between Black and Latino children compared to White children with ASD. Black and Latino boys had significantly higher rates of obesity compared to White boys with ASD, and Latino boys had significantly higher rates of obesity compared to White boys. While these findings are not surprising, it is disturbing. We know that among typically developing populations, Black and Latino children have higher rates of obesity compared to White children (19). There is also robust evidence that children with ASD have higher rates of obesity compared to typically developing peers (5). However, this is the first time to our knowledge that racial and ethnic disparities in obesity have been examined in a sample of boys with ASD, which eliminates residual confounding due to sex.

In order to work towards equity, identifying modifiable and structural factors that may contribute to higher rates of obesity among Black and Latino children with ASD is crucial. Therefore, we examined the relationship of selected social determinants of health to BMIz and obesity among the children in our study and assessed the extent to which any of the social determinants of health explained the observed disparity. We observed strong associations between socioeconomic status, area deprivation, food insecurity, and housing insecurity and obesity among boys with ASD of all races and ethnicities, consistent with findings in TD populations (19, 32–35). In contrast, we did not observe associations with community safety variables such as neighborhood walkability, which had been identified as an obesity risk factor in many studies of TD children (36, 37). Our incongruent findings may reflect the additional safety concerns parents have regarding independent physical activity in their children with ASD, which make typical neighborhood safety characteristics less salient (38, 39).

To assess whether any of the social determinants of health helped to explain disparities in obesity, we first examined whether the determinants differed by race/ethnicity. We found that Black and Latino children scored significantly higher on the ADI, and that Black and Latino parents were more likely to feel that violence as a problem in their neighborhood compared to White parents; these findings parallel the literature on the general US population (21). We did not identify significant racial/ethnic differences in food or housing insecurity or in children's perception of racial/ethnic discrimination; no child reported that they felt discriminated against because of their race, ethnicity, or color. However, these findings should be interpreted with caution given the small sample size and their lack of consistency with other studies. When we examined whether any of the social determinants of health mediated the relationship between race/ethnicity and obesity, only food insecurity among Black autistic boys emerged as a significant mediator of this relationship. Taken together, this suggests that while Black families with an autistic child may not be at greater risk of food insecurity compared to Whites in this sample, such insecurity may play a larger role in diet quality through mechanisms such as food deserts. This would be consistent with the worse area deprivation scores we found among Black families.

Some noteworthy limitations of our study warrant discussion. Autism status was based on parent report on a screening questionnaire rather than a neuropsychological assessment or multi-item measure. This may have resulted in inclusion of boys who would not have met diagnostic criteria. Similarly, the social determinants of health measures we relied upon are subject to misclassification. However, we would not expect any measurement error to differ by race/ethnicity. While the ABCD data is large national study, the number of children identified with ASD is small, limiting the power to detect group differences, especially between Black and Latino boys. A larger sample may allow us to better explain the association of social determinants on obesity among children with ASD for example. Further, because of the small sample and 4-to-1 male to female ratio, the sample did not have an adequate number of girls for a separate analysis.

In conclusion, our findings suggest substantial racial/ethnic disparities exist among children with ASD, mirroring the disparities observed in neurotypical youth. Additional research is needed to better elucidate unique social determinants of obesity risk in these populations and further study is needed in girls with ASD. Given the myriad health consequences of obesity for all children, more attention needs to be paid to the maintenance of healthy weight of Latino and Black boys with ASD and to identifying and addressing modifiable factors that are associated with unhealthy weight among children with ASD from Black and Latino communities.

## Data Availability

Publicly available datasets were analyzed in this study. Data are available on The National Institute of Mental Health Data Archive (NDA) website.
